# A quantum-inspired probabilistic prime factorization based on virtually connected Boltzmann machine and probabilistic annealing

**DOI:** 10.1038/s41598-023-43054-5

**Published:** 2023-09-27

**Authors:** Hyundo Jung, Hyunjin Kim, Woojin Lee, Jinwoo Jeon, Yohan Choi, Taehyeong Park, Chulwoo Kim

**Affiliations:** Korea University, Seoul, Korea

**Keywords:** Electrical and electronic engineering, Computational science

## Abstract

Probabilistic computing has been introduced to operate functional networks using a probabilistic bit (p-bit), broadening the computational abilities in non-deterministic polynomial searching operations. However, previous developments have focused on emulating the operation of quantum computers similarly, implementing every p-bit with large weight-sum matrix multiplication blocks and requiring tens of times more p-bits than semiprime bits. In addition, operations based on a conventional simulated annealing scheme required a large number of sampling operations, which deteriorated the performance of the Ising machines. Here we introduce a prime factorization machine with a virtually connected Boltzmann machine and probabilistic annealing method, which are designed to reduce the hardware complexity and number of sampling operations. From 10-bit to 64-bit prime factorizations were performed, and the machine offers up to 1.2 × 10^8^ times improvement in the number of sampling operations compared with previous factorization machines, with a 22-fold smaller hardware resource.

## Introduction

Deterministic computers have been developed to enhance computing power using nanoscale transistors. However, despite the increasing demand for solving complex combinatorial optimization problems (COPs), deterministic computers perform slow and inefficient search operations^1^, and the process-scaling of transistors has been reaching its limit^2^. Quantum computers have been introduced to rapidly solve non-deterministic polynomial (NP)-hard problems^3–5^. However, general-purpose quantum computers require a large number of qubits with high precision, which is unlikely to be physically implemented in the near future. Thus, quantum annealers^6–9,43–48^ have been introduced to reduce the computation time of real-world COPs for the practical implementation of quantum computers. Nevertheless, many-body interactions in adiabatic quantum computing^49–51^ require a large number of physical qubits with complex hardware connections.

As a result of the above issues, probabilistic computing^10–24^, which is realized by using complementary metal–oxide–semiconductor (CMOS) technology, has been proposed to cost-effectively replace quantum annealers, especially for solving complex COP as factorization calculations^10–12,52^. These CMOS-based Ising machines have improved the factorization speed by four^11^ and six^12^ orders above central processing units. However, the hardware complexity and computation time of previous factorization machines sharply increase as the number of total probabilistic bits (p-bits) increases. Therefore, a novel algorithm and its hardware implementation are still required for practical applications.

**Figure 1:**
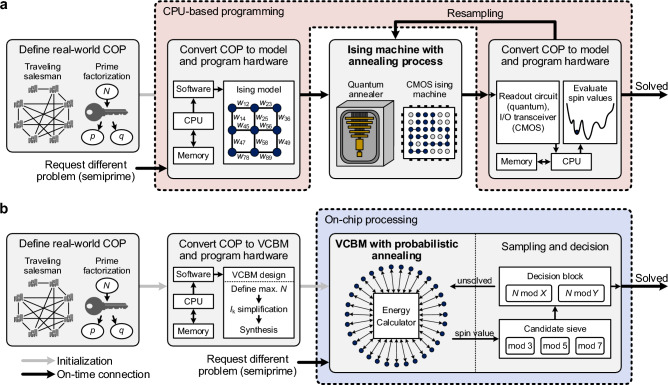
Workflows of previous Ising machines and proposed probabilistic annealer. (**a**), For solving the real-world COP, the previous studies employed CPU-based programming to control the Ising machines. (**b**), In the current study, we propose the VCBM that improves the functionality of the probabilistic machine, and on-chip processing units that further accelerate the speed of prime factorization.

In this study, we propose a virtually connected Boltzmann machine (VCBM) that achieves less hardware complexity than state-of-the-art Ising machines. Figure 1 shows a comparison between the workflows of the previous Ising machines and the proposed probabilistic annealer. To solve real-world COPs with annealing processors, the COPs are converted to target hardware using software programs. This initialization operation is also conducted in our probabilistic annealer, but the energy calculator in the VCBM eliminates the need for the re-programming process of conventional Ising machines to solve different semiprimes. In order to further accelerate the performance of the factorization machine with on-chip processing, we employed three modulo operators (divide *X* and *Y* candidates by 3, 5, and 7) preceded in each case by a sieve to determine the best candidate (hereafter termed a “candidate sieve”) and a decision block that consists of two modulo operators (divide the semiprime by best candidate). In this work, we also introduce a probabilistic annealing method, which reduces the number of sampling operations of the Boltzmann machine. To demonstrate its potential to the community, results for up to 64-bit factorization were produced using a field-programmable gate array (FPGA).

### Virtually connected Boltzmann machine

**Figure 2:**
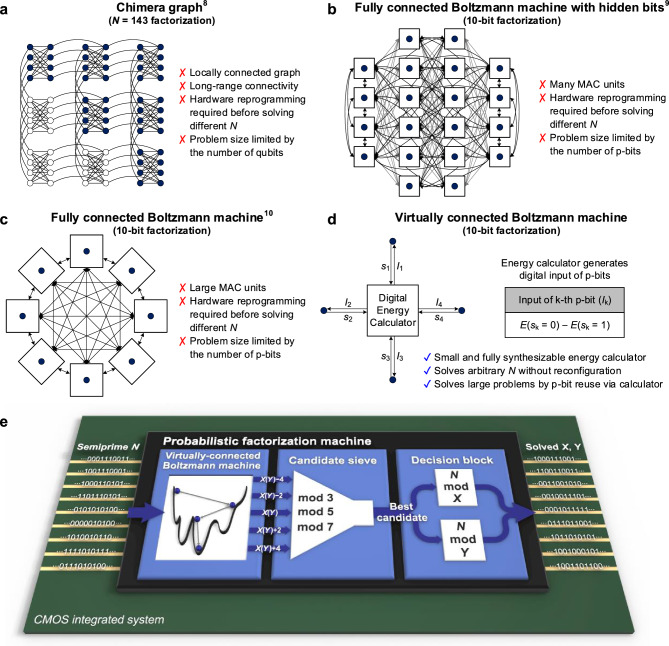
Demonstration of the VCBM and developed factorization machine. (**a**), Architecture of the D-Wave 2000Q quantum annealer^8^. The locally connected hardware has limitations for solving complex COPs. (**b, c, d**), Architectures of graph-model-based factorization machines. Circles represent p-bits, and squares represent the approximate hardware area of the digital logic. **(b)** The hardware cost of the general Boltzmann machine can be reduced by replacing the 3-body and 4-body terms with hidden p-bits^9^. However, hidden p-bits also increase hardware complexity. (**c**) In the graph model, p-bits of the general Boltzmann machine require a large area of spin-weight matrix multiplication logic^10^. (**d**) The architecture of the proposed VCBM. The digital input of p-bits can be calculated using the term *E*(*s*_k_ = 0) – *E*(*s*_k_ = 1). Thus, the energy calculator generates p-bit inputs without spin-weight matrix multiplication. (**e**), Architecture of our probabilistic factorization machine. In this work, we implemented a prototype of a 64-bit general-purpose factorization machine using an FPGA.

Figure 2a shows a chimera graph of the quantum annealer, which was used for factorizing 143 in a previous work^8^. This previous work factorized up to 249,919 using a D-Wave 2000Q quantum annealer. Zephyr and Pegasus graphs are also used for implementing D-Wave quantum processing units, but the topologies of quantum annealers consist of sparsely connected hardware connections between qubits. Thus, these graphs require further complex hardware connections between qubits for embedding a fully connected graph model to solve complex COPs. In addition, the quantum annealer has difficulty increasing the number of spins owing to the long-range connection between qubits during the annealing process.

The Boltzmann machine^25–27^ is a type of constraint satisfaction network that uses its probabilistic dynamics to lead a system to ground state. Figure 2b shows the Boltzmann machine hardware with hidden bits that represent 3-body or 4-body terms^9^. However, a large number of hidden bits increases the number of hardware connections to the fourth power of *N* bits. To reduce the hardware complexity caused by hidden p-bits, the hardware can be implemented with spin-weight matrix calculation circuits that conduct the calculation of the 3-body and 4-body terms of Ising model^10^, as shown in Fig. 2c. Nevertheless, the hardware requires large multiply-accumulate (MAC) units, and the weight input data of the units should be fully programmable to solve various searching problems (see Methods for details). In 2022, restricted Boltzmann machine (RBM)^28^-based probabilistic computing was introduced^11^, but the large number of hardware connections in the RBM still limits the application of these machines in large-scale general-purpose probabilistic computers. Therefore, previous Ising machines consumed significant areas, hardware design times, and routing resources. Moreover, these machines require re-programming using an additional deterministic computer that formulates the hardware connections of the Ising machine before solving problems^10–24^. Furthermore, because these machines use one-to-one corresponding hardware to their graph models, the size of the problem that can be solved is strictly limited by the number of p-bits.

Figure 2d shows the high-level concept of the VCBM. In the general Boltzmann machine, the input value of the k-th p-bit (*I*_k_) represents every connected interaction in the system to the visible p-bit. However, the computational power of CMOS digital circuits can be used to generate virtual connections of each visible p-bit. Thus, in our machine, the energy calculator is employed to calculate the digital input of each p-bit, replacing the need for a large and complex chain network composed of digital blocks or hidden p-bits. Therefore, the number of input and output signals of the energy calculator are greatly reduced and the energy calculator can be fully synthesized with simple digital codes, both of which dramatically decrease the hardware complexity above previous probabilistic machines. Moreover, the energy calculator can operate hardware-efficiently by reusing the p-bits to solve large problems or dividing the p-bits into groups to solve many small problems. In addition, since the VCBM uses an equivalent probability equation of input to p-bits as the general Boltzmann machine, the VCBM represents an all-to-all connected Boltzmann machine, which is suitable for solving large and complex COPs.

Figure 2e shows the hardware architecture of our implemented factorization machine. The proposed VCBM enables our machine to continuously perform from 10-bit to 64-bit factorization for various *N* numbers without additional hardware connection re-programming. For conducting the prime factorization operations, the energy calculator generates *I*_k_ as,

1 $${\text{Ik }} = 2^{{3 + k - 2n}} ({\text{N}} - {\text{XY}}){\text{Y }} \pm 2^{{1 + 2k - 2n}} {\text{Y}}^{2} .$$

Thus, the energy calculator conducts a single calculation of (*N*–*XY*)*Y* and *Y*^2^, and generates multiple *I*_k_ values by simply bit-shifting the calculated (*N*–*XY*)*Y* and *Y*^2^ values (Detailed information on the derivation is provided in Methods).

Compared to the quantum annealers, we think that the major advantage of CMOS-based annealers is their robust digital operations within a single chip at room temperature. Thus, we propose a probabilistic factorization machine architecture accelerated by using the Boltzmann machine along with the on-chip processing units. Considering that the Boltzmann machine frequently generates a high-quality output that is close to the global ground state, a set of nearby numbers of the output of the machine output also have a high probability as an answer. Thus, we inserted a candidate sieve between the machine and the decision block to choose the candidate that is close to the output of the Boltzmann machine but cannot be divided by 3, 5, and 7 (best candidate) with small area consumption. Then, two modulo operators of the decision block, which are pipelined to operate in two cycles to reduce the critical-path delay, check whether *X* or *Y* is the factor of *N*. Therefore, the decision block determines the end of the factorization operation. For factorizing up to 64-bit numbers, the machine uses 31 p-bits that are implemented based on lookup tables (LUTs)^11,29^ of the FPGA hardware. Detailed information on the FPGA implementation is provided in Methods.

### Probabilistic annealing

**Figure 3:**
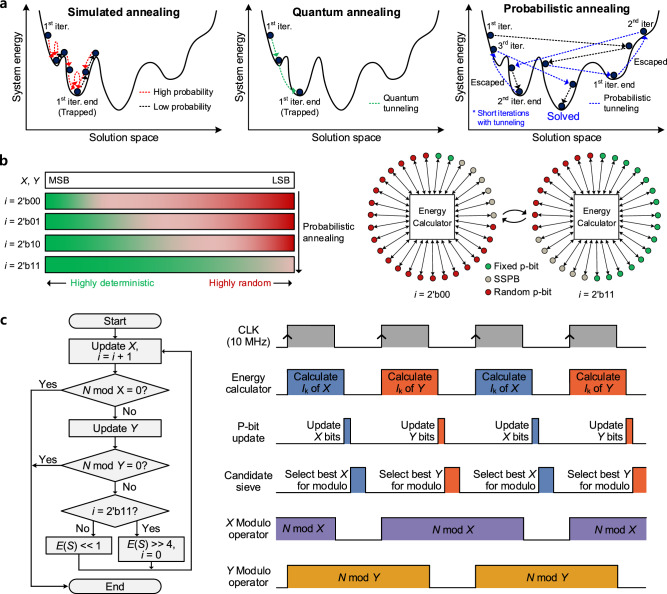
Demonstration of probabilistic annealing. (**a**), Conceptual diagram of state transitions during annealing processes. In general, sequential updating makes it difficult for the system to escape from a local minimum. Quantum annealer employs the quantum annealing method to tunnel the energy barriers, targeting to reach the minimum energy state during the annealing process. However, sampling operation by the readout circuit is required to analyze the system energy of the final state. The probabilistic annealing of the current work was designed to accelerate the system convergence to a minimum with parallel updating and dynamic SSPB control. Upon completion of 8 sampling operations (in which the number 8 is constant for every factorization operation in this work), the system restarts a searching iteration from a higher energy state with high probability, until achieving the solution to the problem. (**b**), High-level concept diagram of the probabilistic annealing process. The number of fixed p-bits and random p-bits are not always equivalent as shown in the figure, since the p-bits change stochastically by their current energy state. **c,** Flowchart and operating sequence of the factorization machine. This machine is designed to employ a decision block (*X* and *Y* modulo operators) for determining the completion of factorization when *N* mod *X* or *N* mod *Y* becomes 0. The operation of the candidate sieve is conducted after the p-bit update (omitted in the flowchart for simplicity).

To further improve the factorization operation of the machine, we developed a probabilistic annealing process. The probabilistic annealing consists of performing parallel updates and controlling dynamic system-significant p-bit (SSPB). In previous works, the Ising machines reached the global minimum with sequential updating^10,14–24^ or parallel updating^11–13^, as shown in Fig. 3a. When a fully connected Ising machine is operated with the simulated annealing process, a single p-bit is updated sequentially due to its hardware connection. Also, a single p-bit update is performed to have lower energy state at each state with a high probability, making the system converge toward the minimum energy state. Although the probability of a p-bit provides the system a chance to increase the system energy, consecutive low-probability decisions should be conducted to climb the energy barriers. Thus, the sequential updating rather traps the system in a local minimum. On the other hand, parallel update machines were implemented for achieving shorter problem solving, using RBM^2^, sparse Ising model^12^, and stochastic cellular automata (SCA)^13,30^. However, the RBM and sparse Ising model require pre-training before each search operation. The SCA machine has been reported to solve max-cut problems with a level of quality equivalent to those of sequential update machines, but the machine converges the system energy through slow logarithmic cooling along with penalty control, which rather fixes the system to a specific wrong solution in factorization problems. Moreover, previous parallel-updating machines did not achieve a computational complexity advantage over those using the sequential update method, which means they shortened the operation time at the expense of increasing the hardware area. In quantum annealers, the system tunnels through the energy barriers to reach the minimum energy state. However, the number of required sampling operations also increases as the size of the system increases when solving complex COPs, due to the decreased percentage of answers caused by noise and control errors^8,44^. Nevertheless, the system energy is determined only after the sampling operation by the readout circuit in the final state, which worsens the hardware cost of computation in high-number factorization problems.

Therefore, we introduce the probabilistic annealing to make the system rapidly converge to a minimum. In contrast to sequential updating, the simultaneous changing of p-bits would occasionally move system energy through an energy barrier with a single update (probabilistic tunneling). However, simultaneous updating of independent p-bits increases the divergence of the system energy. Considering an interaction between a system and p-bit, when the strength of the interaction is increased, the p-bit would be strongly fixed to 0 or 1. Otherwise, when the interaction is weak, the output of the p-bit would have an approximately 50% probability of being 0 or 1. If a p-bit has an adequate amount of interaction with the system, the p-bit would become an SSPB and be highly likely to induce the system to decrease its system energy; the digital input of the p-bit is signed 8-bit, thus, the SSPB is defined as p-bits that have digital input between 8’b0000_0000 and 8’b0111_1111 or 8b’1111_1111 and 8’b1000_0000. Therefore, constraining the number of SSPBs to a low value and dynamically changing SSPBs to other p-bits after the update are required to have a system converge rapidly to a ground state.

We implemented dynamic SSPB control with a shift register in the energy calculator along with the cost function^36^ *E*(*S*) = *E*_0_ (*XY*–*N*)^2^. In this work, the coefficient of the cost function (*E*_0_) is 2^3–2*n*^, where *n* represents the number of digital bits of *N*. Since *E*(*S*) is a most-significant-bit (MSB)-dominant function, *E*(*S*) naturally constrains a small number of p-bits to become SSPBs, depending on the current energy state of the system. Then, by using the shift register to left-shift *E*(*S*), the system changes the SSPBs from MSB to a less significant bit of *X* and *Y* during the factorization operation, as shown in Fig. 3b. Therefore, the probabilistic annealing periodically changes SSPBs of *X* and *Y* from the bits close to the MSB to the bits close to the least significant bit (LSB), forcing the system to converge to a minimum rapidly.

Figure 3c shows the operating algorithm of the factorization machine. Considering that *N* is factored into two independent prime numbers, the machine updates *X* and *Y*, targeting to converge *X* and *Y* to different prime numbers. After each update, the candidate sieve conducts the modulo operation by 3, 5, and 7 with four candidates nearby *X* (*X*–2, *X*, *X* + 2, *X* + 4) or *Y* (*Y*–2, *Y*, + 2, *Y* + 4). When *X* is updated, the candidate sieve simultaneously divides *X*–2, *X*, *X* + 2, *X* + 4 by 3, 5, and 7, and choose the best candidate that is not divisible from the sequence of *X*, *X* + 2, *X*–2, and *X* + 4. If all of these candidates are divisible by 3, 5, or 7, the candidate sieve selects *X*–4 as the best candidate because *X*–4 is naturally indivisible by 3, 5, and 7. Since the candidate sieve filters the unwanted *X* and *Y* candidates to be operated with the decision block, increasing the number of modulo operators in the candidate sieve further accelerates the factorization performance. However, in this work, to show the effectiveness of the proposed VCBM architecture, a 64-bit factorization machine was implemented using a relatively small FPGA hardware compared to previous works^11,12^. Thus, the hardware of the machine was designed carefully considering the LUT utilization, and we chose to implement the candidate sieve to be operated with three modulo operators. Therefore, the machine only conducts decision operations on a candidate that cannot be divided by 3, 5, or 7. After a consecutive *X* and *Y* update, the machine operates the left-shift of the cost function *E*(*S*) for conducting the probabilistic annealing. Then, upon completing 8 sampling periods (4 samplings per *X* and *Y* each), the machine resets the bit-shifted *E*(*S*) to the original state, making the system have a high energy state again. Thus, our probabilistic annealing process is not designed to find the answer with a single and long iteration as in previous works, but rather to repeat less-energy-barrier-constrained searching iterations toward a minimum with 8 sampling periods.

In contrast to previously developed factorization machines requiring static random-access memory (SRAM) to store weights and biases^2^ or use of the MATLAB program to multiply input weights and inverse temperature online^3^, the probabilistic annealing does not have to optimize the weight values of the factorization machine through pre-training and does not have to calculate the complicated temperature equation of the system. Therefore, this work fully conducts operations with synthesized digital gates in an FPGA, achieving a dramatic reduction in hardware complexity.

### Experiment

**Figure 4:**
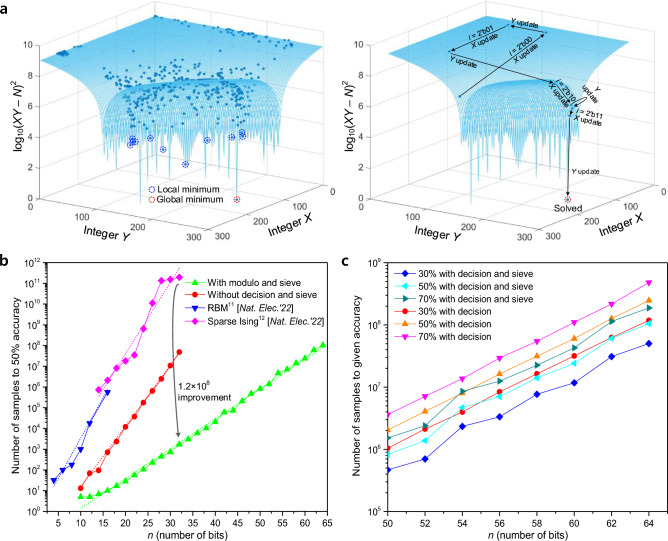
Performance of the machine for factorization compared to previous parallel update factorization machines. (**a)**, 3-dimensional energy landscape of the *N* = 33,499 (241 × 139) factorization and an example of energy state during a single factorization operation. The energy states at the end of each iteration (left side) show that the probabilistic annealing enables frequent movement to the local minimums. In addition, the energy states of the last iteration (right side) show that the annealing process decreases the energy state dramatically and finally leads to the global ground. (**b**), Results of 4-bit to 64-bit factorization measurements of the previous works^11,12^ and the current work are shown. For a fair comparison between annealing schemes, performances without on-chip processing units are also shown. We repeated the experiment 1,000 times, and the number of sampling operations of the conventional sparse Ising machine^12^ is calculated using its 100% time-to-solution results. As shown in the graph, the probabilistic annealing achieved a performance improvement of up to 1.4 × 10^4^ times at 30-bit factorization, and the digitally accelerated architecture reduced the number of sampling operations by 1.2 × 10^8^ times than the previous work^12^ at 32-bit factorization. (**c**), Detailed factorization results from 50-bit to 64-bit with decision block are shown. Using the candidate sieve reduced the number of sampling operations at 50% accuracy by up to 66% (66% reduced at 52-bit factorization) with a small hardware cost. Detailed information about the sampling frequencies of these machines is provided in Methods.

Figure 4a shows the 3-dimensional energy landscape of the *N* = 33,499 factorization and a simulated example of the system movement during our probabilistic annealing process. To clearly show the process of the probabilistic annealing method, we simulated the factorization machine operating without on-chip processing units (candidate sieve and modulo operator) and finished the factorization only when *XY* = *N*. The left-hand side of Fig. 4a illustrates the energy states at the end of each iteration. Because the factorization problem is a complex COP, the landscape has a large number of local minimums. Since the proposed machine searches for the global ground state, the results show that the system frequently reaches minimum energy states. However, this trace with multiple minimum states of the proposed machine also shows that the probabilistic annealing enables the machine to escape from the local minimum states. The right-hand side of Fig. 4a shows the system movement during the last iteration. The results also show that our machine increases its energy at the start of the iteration (*i* = 2’b00 state). Contrary to the conventional simulated annealing process, the system energy decreases toward the global ground state, not the nearest local minimum state, which resulted from the probabilistic annealing process.

Figure 4b shows the results of the measurements in the current work compared with those of previous works. The measurement results show that the probabilistic annealing reduces the number of factorization operations by up to 1.4 × 10^4^ times, compared to previous parallel update machines^11,12^. Moreover, when we employed the candidate sieve and decision block for the factorization operations, there was an 1.2 × 10^8^ times reduction in the number of sampling operations at 32-bit factorization compared to the previous work^12^.

Figure 4c shows a graph of the detailed performance results using the candidate sieve and decision block. As seen in this graph, the use of the candidate sieve decreased the number of sampling operations by up to 66% at 50% accuracy; the number of samples at 50% accuracy represents the measured number of samples that can factorize 500 out of 1,000 experiments. Since the critical path delay of our machine is the operation of the decision block, adding the candidate sieve increases only 1.56 ns of additional operation time per sampling period. The sieve costs 1,466 (3.82% of used) LUTs, thus, we demonstrate that the candidate sieve increased factorization performance with a low amount of hardware consumption. Time-domain measurement results of our factorization machine are shown in Supplementary Fig. .

Table 1 summarS1izes the performances of state-of-the-art Ising-model-based factorization machines. Due to the deployment of the VCBM, our work requires the fewest p-bits and is implemented without the MAC unit for weight and spin multiplications. Therefore, compared to recent works^11,12^, we employed a relatively cost-effective FPGA (Xilinx Artix-7) with a smaller amount of programmable logic (PL). Also, contrary to the previous works^11,12^ that require more complex Ising machine hardware for parallel updating, the probabilistic annealing enables parallel updating with the fully connected VCBM. Moreover, due to the high scalability and rapid factorization performance of our machine, we carried out up to 64-bit factorization, which is the highest in the table.

**Table 1:** Comparison of Ising-model-based factorization machines. Here we compare the hardware performances of the state-of-the-art Ising-model-based factorization machines. The RBM work^11^ used quantization retraining for performance enhancement. Compared to the FPGA (Xilinx Virtex UltraScale + VU9P) used in recent works^11,12^, our current work employed a more cost-effective FPGA (Xilinx Artix-7) owing to its small hardware requirements. As shown in the table, our machine factorizes the largest semiprime number with the lowest hardware cost and the smallest number of spins per unit number of semiprime bits.

Platform	Architecture	Annealing	# of LUTs	# of spins	# of bits	Max. factorized number
D-wave 2000Q^8^	Chimera	Quantum	–	1,803	74	249,919
Stochastic MTJ^10^	General Boltzmann	–	–	8	10	945
FPGA^11^	RBM	–	1,182,240	680	16	43,621
FPGA^12^	Sparse Ising	Simulated	1,182,240	2,128	32	4,227,546,633
FPGA (this work)	Virtually connected Boltzmann	Probabilistic	53,200	31	64	14,757,395,536,968,770,247

**Figure 5:**
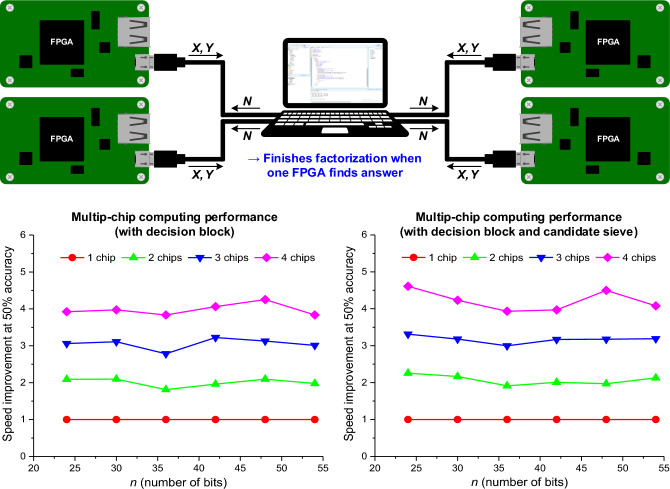
Performance of the machine for multi-chip computing. Measurement environment and measured results of multi-chip factorizations are shown. Four equivalent FPGA boards were connected to a computer and started operation together to factor the equivalent semiprime *N*. The factorization was finished immediately when one of the FPGA boards factorized *N*. The measurements were repeated 1,000 times, and factorization speed improvement (y-axis) represents the normalized number of samples of multi-chip architecture compared to that of the one-chip architecture. Compared to single-chip computing, multi-chip computing with 2, 3, and 4 machines achieved approximately 2.01 × , 3.05 × , and 3.98 × reductions in the number of sampling operations at 50% accuracy (500 solved experiments) with decision block. When the machine with a candidate sieve is employed, the average factorization performance is improved by 2.07 × , 3.17 × , and 4.22 × when using two, three, and four chips, respectively.

Figure 5 shows the test setup and measurement results with four independent FPGAs. In 2021, a multi-chip architecture based on simulated bifurcation^18^ reported up to 1.89 × of computation time reduction by using two chips on a single problem. However, the computation time was reduced by a factor of only 3.32 (i.e., less than 4.00) with four chips, implying that the simulated bifurcation would result in per-chip performance degradation in large-scale multi-chip processor applications. On the contrary, since our VCBM represents the fully connected Boltzmann machine, the average factorization performance is improved by the number of parallel-connected chips. When factorizing with a decision block, our machine improves the average factorization performance by factors of 2.01 × , 3.05 × , and 3.98 × on average when using two, three, and four chips, respectively. Furthermore, our machine with a candidate sieve improves the average factorization performance by factors of 2.07 × , 3.17 × , and 4.22 × when using two, three, and four chips, respectively. Unlike previous annealing processors^18,33^, our machines reduce the operation time without data interaction between each other, thus, the hardware resources can be easily shared and reconfigured according to the size of the problem to be solved. Further detailed measurement results are shown in Supplementary Fig. S2and S3.

### Discussion and outlook

In this work, we have demonstrated a probabilistic factorization machine based on a VCBM and probabilistic annealing scheme. To overcome the hardware complexity of previous Ising machines, we developed the VCBM. The VCBM was designed to update its p-bits using an energy calculator, eliminating the need for complex matrix multiplications. Moreover, unlike previous Ising machines, our machine was designed to factor various semiprime numbers without re-programming hardware connections, enabling the continuous operation of the annealer. As a result, we factorized the highest number with the smallest number of spins and the lowest hardware cost among the state-of-the-art Ising-model-based factorization machines. Also, we proposed the probabilistic annealing method to enable rapid convergence to the global ground state with probabilistic tunneling process. Thus, the machine achieved a factorization performance of up to 1.4 × 10^4^ times faster than previous annealing methods.

Furthermore, we have shown that using on-chip processing can be a key to enhancing the performance of CMOS-based Ising machines. As a result, the number of sampling operations was decreased 1.2 × 10^8^-fold at 32-bit factorization compared to the previous factorization machine^3^. Although the prototype of this work was implemented in an FPGA, we expect that our machine can be implemented using magnetic tunnel junction (MTJ)-based p-bits^34,35^ and a hardware-optimized energy calculator in a single package, allowing solving larger COPs in a short time with lower computing power.

## Methods

### Derivation of the virtually connected Boltzmann machine

In general, in the Ising model^37,38^, the energy function *E*(*S*) of the system with spin vector *S* is

2 $${\text{E}}\left({\text{S}}\right)= - \left( \sum_{\text{i}<\text{j}}{\text{w}}_{\text{ij}}{{\text{s}}}_{\text{i}}{{\text{s}}}_{\text{j}}+\sum_{\text{i}}{{\text{h}}}_{\text{i}}{{\text{s}}}_{\text{i}} \right),$$

where *s*_i_ and *s*_j_ are state of the i-th and j-th spin, *w*_ij_ is the weight of the edge between the i-th and j-th spins, and *h*_i_ is a bias term for the i-th spin. Consider defining the probability *p* of the system with status *S* as^25–27,39^

3 $${\text{p}}({\text{S}}) = \frac{\text{exp}(- \,{\text{E}}({\text{S}}))}{\text{Z}},$$

where *Z* is the sum of all the cases of system energy and given using the equation

4 $$Z = \mathop \sum \limits_{S} \exp ({-} E(S)).$$

Then, in a Boltzmann machine, the lower the energy of state *S*, the higher the probability appears, which means that the system more likely moves to a lower energy state after transitions. Therefore, the energy function is set to have the minimum energy state at the solution state, and the Boltzmann machine would move the system energy toward the solution state. Consider the conditional probability *p*(*s*_k_ = 1│*S*) as the probability that the k-th spin will be updated to 1 in the next transition at state *S*,

5 $$\begin{aligned} p(s_{{\text{k}}} ~ = ~1\left| S \right.)~\left| S \right.)~ = & ~\frac{{p\left( {s_{1} ,~s_{2} ,~ \ldots ~,~s_{{{\text{k}} - 1}} ,~1,~s_{{{\text{k}} + 1}} ,~s_{{{\text{k}} + 2}} ,~ \ldots ~} \right)}}{{p\left( {s_{1} ,~s_{2} ,~ \ldots ~,~s_{{{\text{k}} - 1}} ,~0,~s_{{{\text{k}} + 1}} ,~s_{{{\text{k}} + 2}} ,~ \ldots ~} \right)~ + ~p\left( {s_{1} ,~s_{2} ,~ \ldots ,~s_{{{\text{k}} - 1}} ,~1,~s_{{{\text{k}} + 1}} ,~s_{{{\text{k}} + 2}} ,~ \ldots ~} \right)}} \\ = & \frac{{\exp \left( {{-}~E\left( {s_{1} ,~ \ldots ~,~s_{{{\text{k}} - 1}} ,~1,~s_{{{\text{k}} + 1}} ,~s_{{{\text{k}} + 2}} ,~ \ldots ~} \right)} \right)/~Z}}{{\exp \left( {{-}~E\left( {s_{1} ,~ \ldots ~,~s_{{{\text{k}} - 1}} ,~0,~s_{{{\text{k}} + 1}} ,~s_{{{\text{k}} + 2}} ,~ \ldots ~} \right)} \right)/~Z~ + \exp \left( {{-}~E\left( {s_{1} ,~ \ldots ~,~s_{{{\text{k}} - 1}} ,~1,~s_{{{\text{k}} + 1}} ,~s_{{{\text{k}} + 2}} ,~ \ldots ~} \right)} \right)/~Z}} \\ = & \frac{1}{{1 + \exp \left( {E\left( {s_{1} ,~ \ldots ~,~s_{{{\text{k}} - 1}} ,~1,~s_{{{\text{k}} + 1}} ,~s_{{{\text{k}} + 2}} ,~ \ldots ~} \right)~{-}~E\left( {s_{1} ,~ \ldots ~,~s_{{{\text{k}} - 1}} ,~0,~s_{{{\text{k}} + 1}} ,~s_{{{\text{k}} + 2}} ,~ \ldots ~} \right)} \right)}} \\ \end{aligned}$$

Thus, by defining *E*(*s*_k_ = 1) and *E*(*s*_k_ = 0) as, respectively,

6 $${\text{E}}\left( {{\text{s}}_{{\text{k}}} = 1} \right) = {\text{E}}\left( {{\text{s}}_{1} , \ldots ,{\text{s}}_{{{\text{k}} - 1}} ,1,{\text{s}}_{{{\text{k}} + 1}} ,{\text{s}}_{{{\text{k}} + 2}} , \ldots } \right),$$

7 $${\text{E}}\left( {{\text{s}}_{{\text{k}}} = 0} \right) = {\text{E}}\left( {{\text{s}}_{1} , \ldots ,{\text{s}}_{{{\text{k}} - 1}} ,0,{\text{s}}_{{{\text{k}} + 1}} ,{\text{s}}_{{{\text{k}} + 2}} , \ldots } \right),$$

and by defining *I*_k_ = *E*(*s*_k_ = 0) − *E*(*s*_k_ = 1), the conditional probability becomes

8 $$p(s_{k} = 1\left| S \right.) = \frac{1}{{1 + \exp ({-} I_{k} )}}.$$

Therefore, if p-bits follow the probability of a sigmoid function to the input *I*_k_ value, then the machine would follow the Boltzmann distribution^25–27,39^.

In our work, we utilized the energy function *E*(*S*) = *E*_0_ (*XY*–*N*)^2^, and thus the input value of the k-th bit (*I*_k_) for updating *X* can be simplified as,

9 $$\begin{aligned} {\text{Ik}} = & {\text{E}}({\text{sk}} = 0) {-} E({\text{sk}} = 1) \\ = & {\text{E}}_{0} \left( {{\text{X}}_{k,0} {\text{Y}} {-} {\text{N}}} \right)^{2} {-} E_{0} \left( {{\text{X}}_{k,1} {\text{Y}} {-} {\text{N}}} \right)^{2} \\ = & {\text{E}}_{0} ({\text{X}}_{k,0} {-} X_{k,1} ) (({\text{X}}_{k,0} + {\text{X}}_{k,1} ) {\text{Y}} {-} 2{\text{N}}) {\text{Y}} = 2^{{2 + k{-}2n}} (2{\text{N}} {-} ({\text{X}}_{k,0} + {\text{X}}_{k,1} ) {\text{Y}}) {\text{Y}} \\ = & 2^{{3 + k{-}2n}} ({\text{N}} {-} {\text{XY}}) {\text{Y}} \pm 2^{{1 + 2k{-}2n}} {\text{Y}}^{ 2} \\ \end{aligned}$$

with *n* representing the number of bits of *N*, coefficient *E*_0_ being 2^3–2*n*^, and *X*_k,0_ and *X*_k,1_ representing the *X* values with 0 and 1 at k-th bit, respectively. Therefore, the energy calculator calculates (*N*–*XY*)*Y* and *Y*^2^ only once, and multiplies them by 2^3+k–2*n*^ and 2^1+2k–2*n*^ simply using the shift register. Then, if the k-th bit of *X* (*X*_k_) is 1, the calculator adds these two terms, otherwise, the calculator subtracts them. Therefore, the VCBM represents a fully connected Boltzmann machine without the need to carry out weight-spin multiplication and accumulation operations. Due to the generality of the derivation of the VCBM, the machine can also be applied to other NP-hard problems that the Boltzmann machine can represent.

In VCBM, only (*n* / 2–2) visible p-bits are required since *X* and *Y* are updated in turn. In addition, considering the hardware cost of the (*N*–*XY*)*Y* multiplier, the computational complexity of the VCBM is determined to be O(*n*^3^), where *n* is the number of digital bits of semiprime *N*.

### Hardware cost of previous Boltzmann machines

In hardware-implemented Ising machines, inputs of p-bits are generated by adding a bias term to the sum of the product of the weight and output of connected spins^37^. However, as the size of the problem is increased, the number of required p-bits increases, and accordingly, the number of p-bit connections dramatically increases. Since the computational complexity of digital logic shows the effectiveness of the hardware, we also derived the hardware costs of previous fully connected Boltzmann machines. For a fair comparison, the hardware costs were considered with the machine that has the energy function *E*(*S*) = *E*_0_ (*XY*–*N*)^2^ and factorizes *n*-bit semiprimes with (*n*/2 − 1)-bit *X* and (*n*/2 − 1)-bit *Y*.

In the general Ising model^37^, the energy function* E*(*S*) can be expressed as, ${(}\mathop \sum \limits_{{\text{i}}} {\text{x}}_{{\text{i}}} {2}^{{\text{i}}} { } \cdot \mathop \sum \limits_{{\text{j}}} {\text{y}}_{{\text{j}}} {2}^{{\text{j}}} {\text{{-} N)}}^{{2}}$, where *x*_i_ and *y*_j_ are the i-th and j-th binary bits of *X* and *Y*, respectively^9,39^. Then, this function can be classified into five different terms^10^: an 1-body constant bias term, a 2-body *x*_i_ ∙ *y*_j_ term, a 3-body *x*_i_ ∙ *x*_k_ ∙ *y*_j_ and* x*_i_ ∙ *y*_j_ ∙ *y*_k_ terms, and a 4-body *x*_i_ ∙ *x*_k_ ∙ *y*_j_ ∙ *y*_l_ term. However, for implementing the hardware with simple weight-spin matrix operators, the general Ising model should be expressed without 3-body and 4-body terms^11,12^, as shown in Fig. 2b. Thus, 3 $\left( {\frac{{{\text{n / 2{-}1}}}}{2}} \right)$ hidden p-bits are produced for eliminating 3-body terms, and (*n*/2 − 1)$\left( {\frac{{{\text{n / 2{-}1}}}}{2}} \right)$ hidden p-bits are required to eliminate 4-body terms, as shown in Fig. 2b. Therefore, (n^3^–4n^2^–4n + 16) / 16 hidden p-bits are required for one additional visible p-bit in the general Ising model. Assuming the use of fixed-point weight bits by the machine, 2-body terms of each p-bit require summation operations of the weight-spin matrix proportional to *n*^3^. In conclusion, based on the total number of p-bits being proportional to *n*^4^, the computational complexity of the machine is determined to be *O*(*n*^7^).

The Ising model can also be implemented by using 3-body and 4-body terms to express the energy function without hidden p-bits^1^, as shown in Fig. 2c. Then, the Ising machine requires *n* − 2 visible p-bits for representing *X* and *Y*. However, (*n*/2 − 1)$\left( {\frac{{{\text{n / 2{-}1}}}}{2}} \right)$ 4-body terms are required to be summed for calculating *I*_k_. As a result, each p-bit requires calculations proportional to *n*^3^, making a computational complexity of *O*(*n*^4^).

Moreover, the hardware complexity of previous probabilistic machines dramatically increases during the digital implementation of the annealing process. Due to the precision of weights determining the hardware accuracy of the Ising machine, precise time-dependent weight calculation logic inevitably increases hardware area in large semiprime factorizations. Also, these required weight values vary for factoring different semiprimes^8,9^, making it difficult to implement solely in FPGA hardware. Therefore, the previous work^12^ implemented a weight and inverse temperature multiplication by co-running the MATLAB program online. However, our machine was designed without these complex weight and temperature multiplications, enabling the FPGA to conduct factorization operations itself. The key differences between the machines are summarized in Supplementary Table S1.

### FPGA implementation

We programmed the factorization machine using Verilog-HDL, and coded and simulated using the Xilinx Vivado 2020.2 program. To achieve a shorter operation time, our factorization machine was programmed to conduct sampling operations of every p-bit in one clock cycle. Therefore, the physical computation time of our work can be derived by multiplying the number of sampling operations by the clock frequency (5 MHz for 64-bit).

To compare the sampling frequency with previous work, we also synthesized our machine with a larger FPGA hardware (Xilinx Zynq UltraScale + ZU7EV). Although the hardware is still has approximately 2.3-fold smaller LUTs than the FPGA used in previous works^11,12^ (Xilinx Virtex UltraScale + VU9P), our machine was operated up to 25 MHz for factoring 64-bit. In addition, we synthesized our machine that can factorize up to 32-bit semiprimes with Xilinx Zynq UltraScale + ZU7EV hardware, and the operating frequency was 50 MHz, which was faster than the previous 15 MHz-operating 32-bit factorization machine^12^. Even more, the sampling frequency of our work can be much shortened using conventional approximate calculation techniques.

In previous works, various computing elements have been utilized to realize the p-bits. However, the factorization performance caused by the mismatch between computing elements causes performance degradation^10^, which requires delicate calibration of each p-bit to meet unbiased reference state. Thus, in this work, p-bits were digitally modeled, using the LUT-based sigmoid function and a 48-bit pseudo-random linear-feedback shift register (LFSR)^11,29^. To accurately show the performance of our machine, we did not quantize the integer multiplication operations of the energy calculator. After the integer calculation is finished, the energy calculator generated 8-bit *I*_k_ as a result of the shift register, which consisted of 1 sign bit, 3 integer bits and 4 fractional bits. Thus, the input of the sigmoidal activation function (*I*_k_) ranges from − 8 to 8 in real numbers, and the sigmoid function generates 16-bit output with *I*_k_ input. Thus, the digital comparator compared this 16-bit output of the sigmoid function to the 16-bit output of the LFSR. If the output of the activation was higher, the p-bit was assigned l in the next update, otherwise, it was assigned 0 in the next update. For performing up to 64-bit factorizations, we used a 48-bit LFSR per p-bit that generates 16 pseudo-random bits per clock cycle.

Our 64-bit factorization machine used 38,086 (71.59% of total) LUT resources. However, since the decision block (X and Y modulators) required 4,455 (8.38% of total) LUT resources and the candidate sieve required 1,455 (2.73% of total) LUT resources, these digital circuits accelerated the factorization performance with low hardware cost. We employed a 5 MHz clock for performing up to 64-bit factorizations, and the operation of the candidate sieve generated a critical-path delay. Also, our machine can dynamically operate with a faster clock for a smaller target semiprime *N* by disabling the factorizing operation of unnecessary upper bits of *X* and *Y*.

After a single synthesis of a 64-bit factorization machine, we measured the sampling times by changing the number *N* and the seed number for LFSRs. For practical purposes, our machine generated seed numbers of LFSRs internally, using a single 32-bit seed number input from the computer. We designed an Advanced eXtensible Interface (AXI) IP block to transmit two input data (a 64-bit subprime *N* and 32-bit seed number) and a System Integrated Logic Analyzer (System ILA) IP block to read three output data (31-bit *X*, 31-bit *Y*, and 64-bit operation time). The processor system blocks were operated with a 25 MHz clock, while the VCBM factorization IP block and other blocks were operated with a 5 MHz clock. The Vitis 2020.2 program was employed to process the read and write data of the FPGA via the USB JTAG interface.

### Model cross-validation

We implemented and tested the equivalent Boltzmann machine using MATLAB and Simulink to determine the accuracy of the measurements. From 10-bit to 32-bit semiprime numbers were tested 1,000 times each, and a comparison with the FPGA measurement results is shown in Supplementary Fig. S4. We used a Mersenne Twister(MT)-based uniform random generator model (MT19937ar)^41^, which has a period of a sequence of 2^19937^ – 1, instead of the 48-bit LFSR in p-bit. Since the FPGA hardware is programmed to initialize the *X* and *Y* to start with the deterministic value, the factorization at low-number bits require more samples than those of the MATLAB simulation results. However, when factorizing above 24-bit, the FPGA experiments involved only a ± 7% difference in the number of samples. Thus, we verified that the factorization machine is properly implemented in hardware with sufficient randomness.

### Comparison between probabilistic annealing and simulated annealing process

We also implemented and simulated 50,851 (16-bit) factorization with the VCBM architecture and conventional simulated annealing process. The inverse temperature of the simulated annealing process increased by 1.125 times after updating every 14 p-bits^31,42^, and the final state of the system was determined after 2,100 sampling operations. Supplementary Fig. S5 shows that the *E*(*S*) of the VCBM decreases over time and converges to certain states due to the decreased temperature by the simulated annealing process. However, since the semiprime factorization has many local minimum states around a single global minimum state, the *E*(*S*) of the system frequently converges to local minimum states. We simulated the system for 1,000 iterations, but *E*(*S*) converged to the global minimum state after only 56 iterations. Assuming the machine operates until finding the answer, this result means that 2,100 sampling operations are required with the simulated annealing process for achieving 5.6% accuracy. However, the probabilistic annealing converges rapidly toward the global minimum state at every 8 sampling operations, making the VCBM require only 710 sampling operations to solve equivalent problem with 50% accuracy. Therefore, this work shows faster factorization compared with previous simulated annealing-based factorization machines.

### Supplementary Information

Supplementary Information.

## Data Availability

The data to reproduce the figures within this work is available from the corresponding authors upon reasonable request.

## Code availability

The FPGA and MATLAB codes used in this work have been deposited in a public Github repository (https://github.com/HyundoJung/VCBM_PA.git).
