# Supplementary material for: A quantum-inspired probabilistic prime factorization based on virtually connected Boltzmann machine and probabilistic annealing
Source: Sci Rep. 2023 Sep 27;13:16186. doi: 10.1038/s41598-023-43054-5 (PMC10533543; doi:10.1038/s41598-023-43054-5)
Supplement: Supplementary file 1 — Supplementary Information. [file 41598_2023_43054_MOESM1_ESM.pdf]

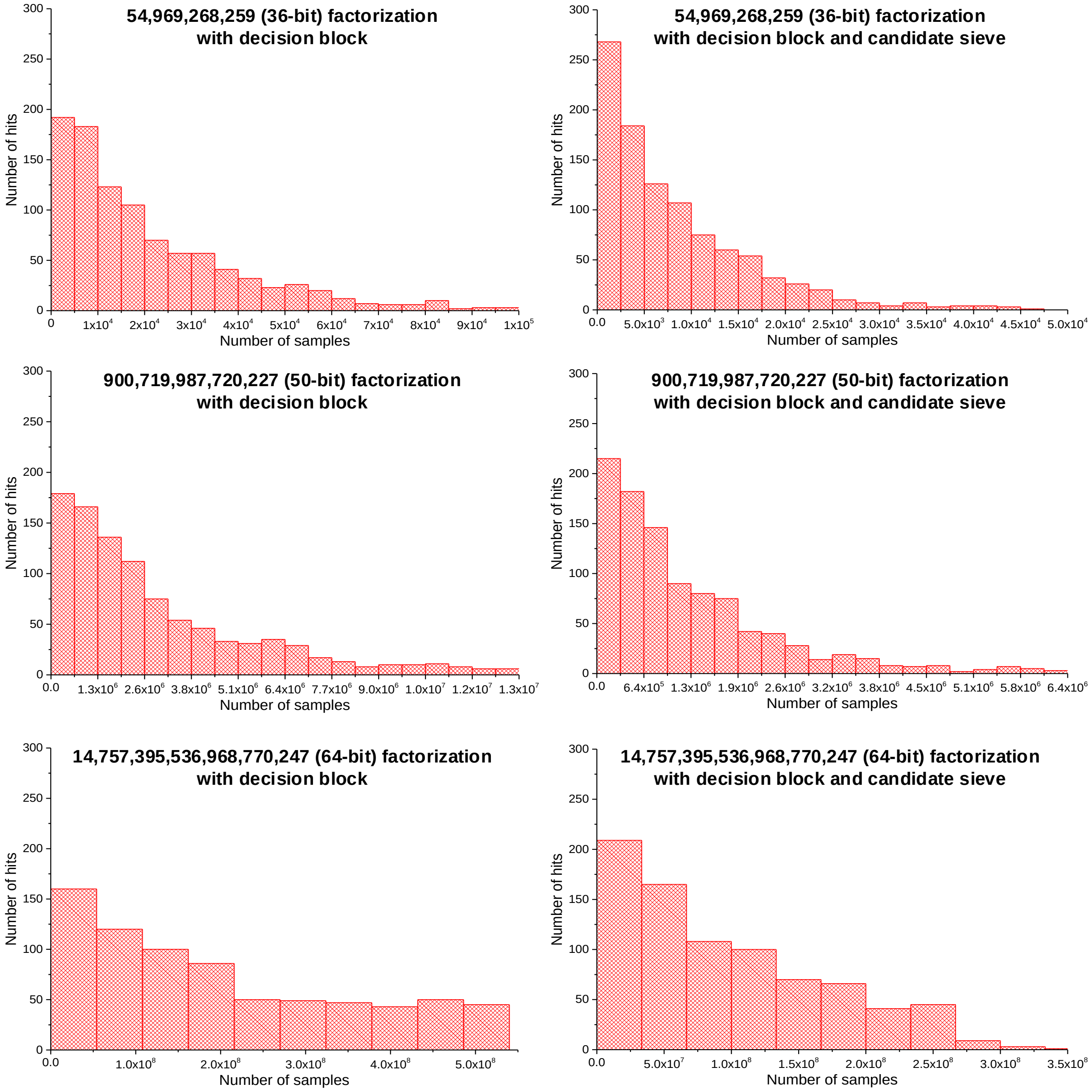

Supplementary Figure S1 | Time-domain histogram of factorizations. The time-domain factorization experiment results are shown for further analysis. Our machine was experimented with the candidate sieve and decision block, and the histograms show that most experiments finished earlier than the middle point of x-axis, due to the Boltzmann distribution.

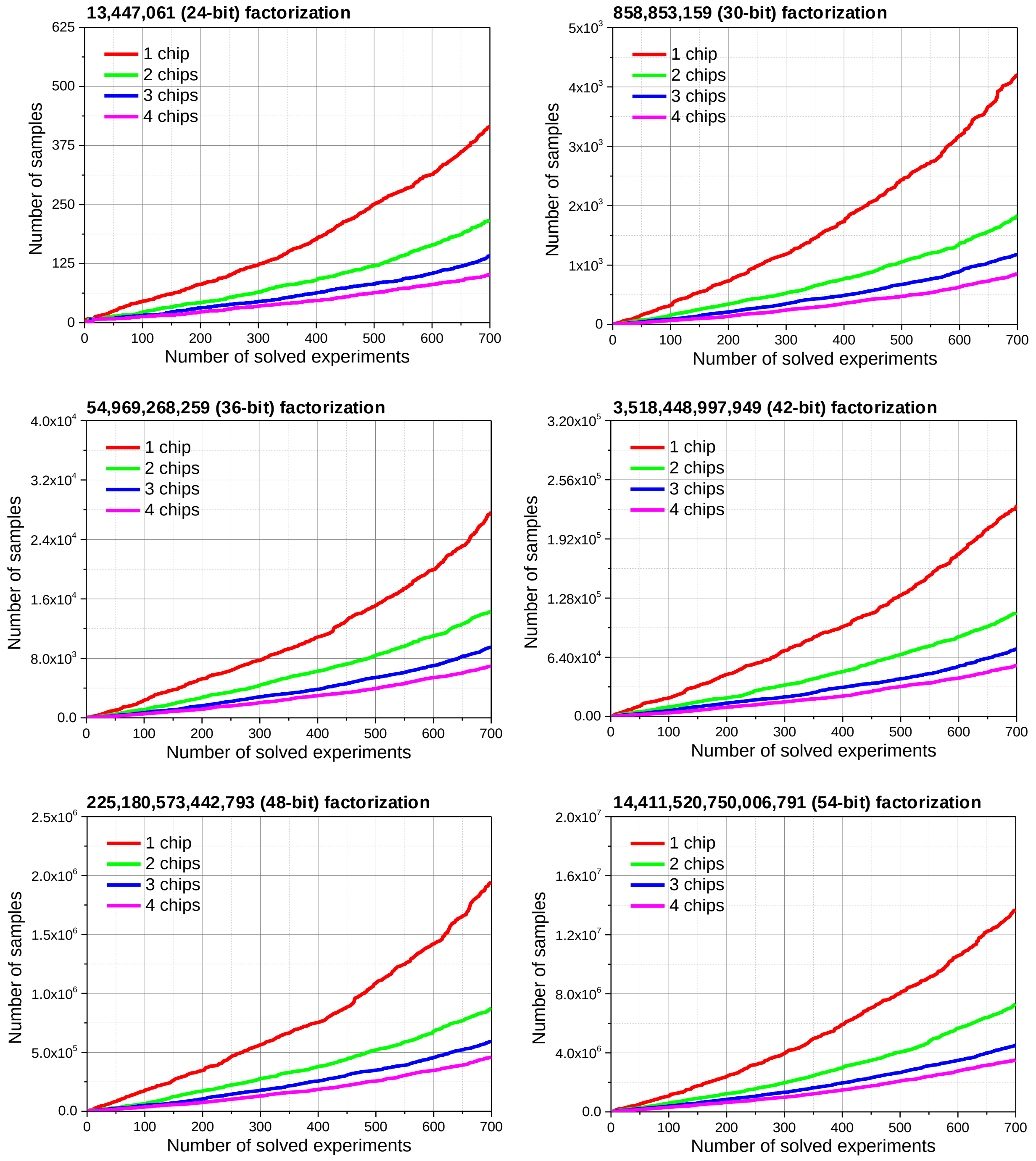

Supplementary Figure S2 | Number of required samples in multi-chip computations with the decision block and without the candidate sieve. The cumulative factorization results are shown for analyzing the performance of the multi-chip computation. Our machine was found to improve the factorization performance by approximately 2×, 3×, and 4× when using two, three, and four chips, respectively.

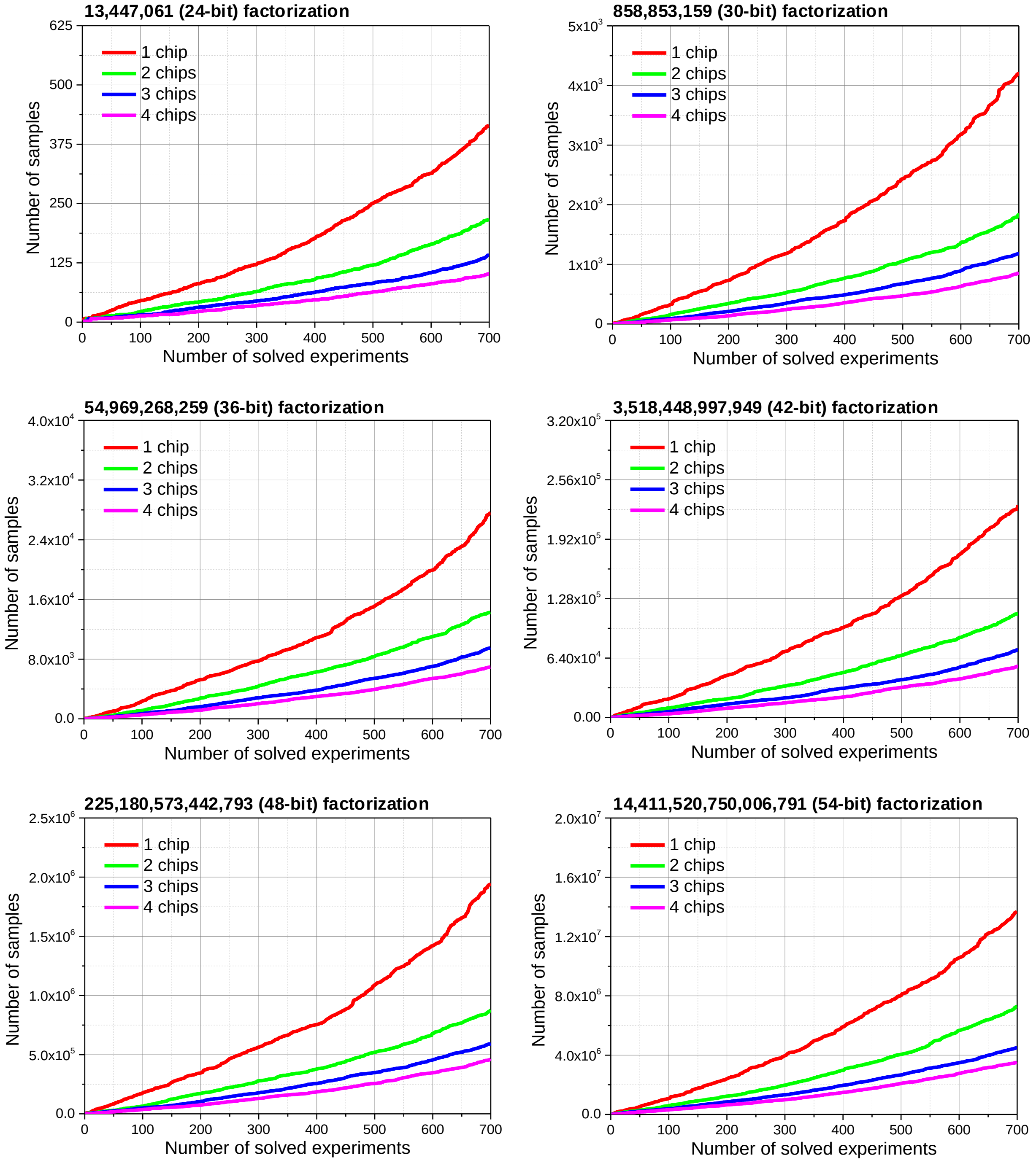

Supplementary Figure S3 | Number of required samples in multi-chip computations with the candidate sieve and decision block. Additional cumulative factorization results are shown for analyzing the performance of the multi-chip computation with the candidate sieve and decision block. Our machine with candidate sieve also improves the factorization performance by approximately 2×, 3×, and 4× when using two, three, and four chips, respectively.

**Platform**	**Quantum Annealer**	**Ising machine (conventional)**	**Probabilistic Annealer (this work)**
Temperature	✗ (15 mK)	✓ (300 K)	✓ (300 K)
# of sampling	✓ (quantum annealing)	✗ (simulated annealing)	✓ (probabilistic annealing)
Annealing time	✓ (quantum-based)	✗ (CMOS-based)	✗ (CMOS-based)
Processing time	✗ (programming)	✗ (programming)	✓ (on-chip processing)
Functionality	✗ (programming per request)	✗ (programming per request)	✓ (energy calculator)

Supplementary Table S1 | Comparison between quantum annealer, conventional Ising machines, and this work. Here we compare the characteristics of the annealing machines. As shown in the table, our machine requires a small number of sampling operations owing to the probabilistic annealing scheme. In addition, the proposed on-chip processing units make the probabilistic annealer achieve high functionality, factorizing from 10-bit to 64-bit arbitrary semiprime with a single hardware synthesis.

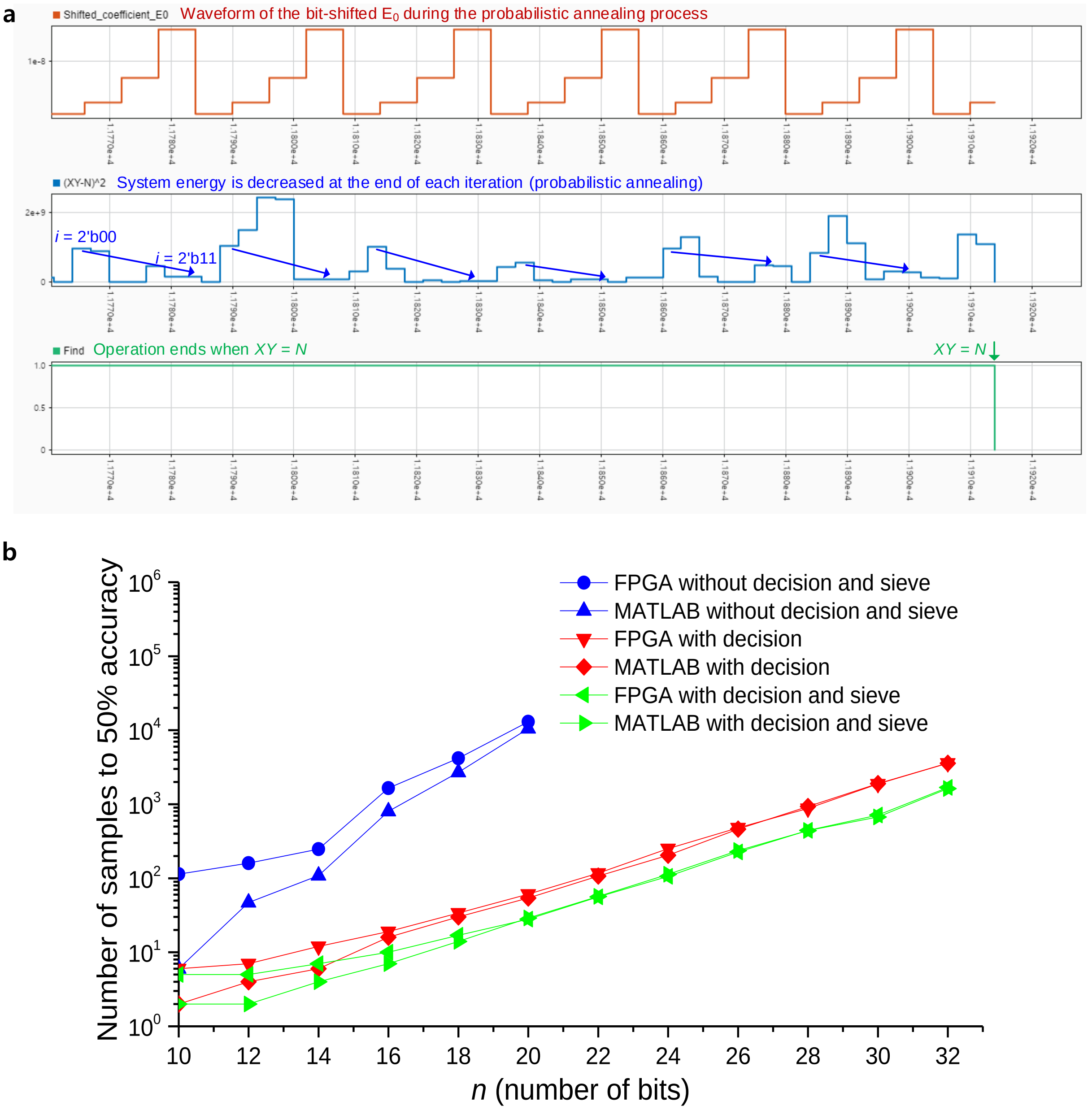

Supplementary Figure S4 | MATLAB Simulink simulation results compared to those of the FPGA experiments. a, Simulated waveform of 16-bit factorization operation without decision block and candidate sieve using MATLAB Simulink. The Simulink model was designed to finish the factorization after the decision block determines the end of the operation. **b,** Results of 10-bit to 32-bit factorizations with the modulo operator when using MATLAB Simulink simulations compared to those obtained from FPGA experiments. For more exact model cross-validation, we tested semiprimes equivalent to those used in FPGA experiments.

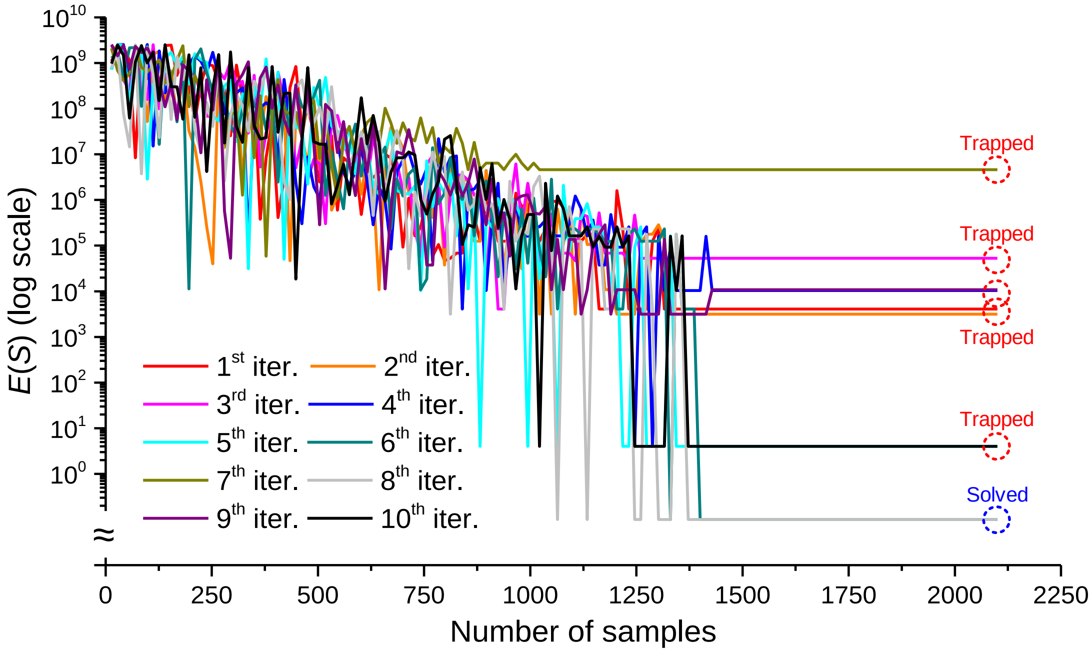

Supplementary Figure S5 | Simulation results of 16-bit factorization with conventional simulated annealing using VCBM hardware. The energy graphs of ten iterations during 16-bit semiprime (50,851) factorization are shown. Since the p-bits of VCBM follows the Boltzmann distribution, the $E(S)$ decreases as the temperature decreases over time. However, since the semiprime factorization has many local minimum states around a single global minimum state and only a single p-bit is updated with the simulated annealing process, the system energy of eight iterations is trapped at local minimum states even after the annealing process.”
